# Myotilin gene duplication causing late‐onset myotilinopathy

**DOI:** 10.1111/ene.70029

**Published:** 2025-01-05

**Authors:** Marco Spinazzi, Marco Savarese, Franck Letournel, Lydia Sagath, Florence Manero, Agnès Guichet, Alexander Hoischen, Corinne Metay, Julien Gouju, Bjarne Udd

**Affiliations:** ^1^ Neuromuscular Reference Center, Department of Neurology CHU d'ANGERS/ INSERM U1083 Angers France; ^2^ Neurobiology and Neuropathology Unit, Department of Pathology CHU d'Angers Angers France; ^3^ Folkhalsan Research Center Helsinki Finland; ^4^ Department of Human Genetics, Research Institute for Medical Innovation Radboud University Medical Center Nijmegen Netherlands; ^5^ SCIAM, SFR ICAT University of Angers Angers France; ^6^ Service de Génétique CHU—CNRS 6015/INSERM U1083—Université d'Angers Angers France; ^7^ Department of Internal Medicine, Radboud Expertise Center for Immunodeficiency and Autoinflammation and Radboud Center for Infectious Disease (RCI) Radboud University Medical Center Nijmegen Netherlands; ^8^ APHP, GH Pitie‐Salpetriere, Molecular and Cellular Cardiogenetics and Myogenetics UF, Chromosomic and Molecular Genetic Center and INSERM UMRS 974 Institute of Myology Paris France; ^9^ Tampere Neuromuscular Reference Center Tampere University Hospital Tampere Finland

**Keywords:** distal myopathy, duplication, late‐onset myopathy, long‐read sequencing, muscle hypertrophy, myofibrillar myopathy, myotilin

## Abstract

**Background:**

myotilinopathy is a very rare inherited muscle disease that belongs to the group of myofibrillar myopathies. These diseases share a common alteration of the sarcomere organization at the level of the Z disk resulting in pathological protein aggregation, autophagic abnormalities, and ultimately muscle degeneration. Most reported cases are due to dominant missense mutations in the *MYOT* gene, two of which are largely recurrent.

**Methods:**

We describe the clinical, radiological, pathological, and molecular analysis including long‐read sequencing of a family affected by late‐onset dominant proximodistal myopathy and muscle hypertrophy.

**Results:**

We identified a duplication of the entire *MYOT* gene as the molecular cause of late‐onset‐myotilinopapthy with typical clinical and pathological features.

**Conclusions:**

This study expands the molecular spectrum of myotilinopathy and highlights the use of long‐read sequencing in the diagnosis of genetic neurological diseases caused by duplications and genomic structural variants. Myotilinopathy as well as other myofibrillar and distal myopathies should be considered in the differential diagnosis of patients affected by distal muscle weakness, even when presenting at an old age.

## INTRODUCTION

Myofibrillar myopathies are genetically heterogenous rare diseases of skeletal muscles sharing a common pathology process of myofibrillar degradation at the level of Z disk, leading to the accumulation of proteins derived from degradation of myofibrils associated with compensatory activation of autophagic processes [1]. Myotilinopathies are a subset of myofibrillar myopathies (MFM), characterized by the presence of mutations in the *MYOT* gene, which encodes the protein myotilin, which is mainly expressed in the Z‐disk of striated muscle. Inheritance of this disease is mostly autosomal dominant. Myotilin is a 57 kDa protein that plays a crucial role in maintaining the structural integrity of the sarcomeres, the basic unit of muscle contraction. It is predominantly expressed in skeletal muscle and to a lesser extent in cardiac muscle. Myotilin interacts with other sarcomeric proteins such as α‐actinin, filamin C, and actin, contributing to the stabilization of the Z‐disc and the overall architecture of muscle fibers [2]. Clinically it is characterized by progressive muscle weakness starting usually in distal leg muscles and rarely more proximally. Sometimes heart muscle may be involved leading to cardiomyopathy. Peripheral neuropathy has been reported in some patients although with unsettled causality [3]. The diagnosis relies on the typical muscle pathology [4] and the identification of pathogenic mutations in the *MYOT* gene. Pathogenic mutations so far reported include mostly single nucleotide substitutions, typically missense mutations, more rarely small deletions leading to frameshift [5], or a recently reported inframe deletion [6]. In this study, we report a family with a duplication of the whole *MYOT* gene and late‐onset myotilinopathy.

### Patients and methods

We studied two affected patients in a French family. Detailed clinical investigations were carried out including pedigree analysis, detailed neuromuscular examination, serum creatinine kinase (CPK) level assessment, electrophysiological studies, and lower limb muscle MRI.

Muscle pathology was conducted on frozen muscle sections according to standard methods. For immunohistochemistry, the following antibodies were used anti‐myotilin mouse monoclonal antibody (Leica Biosystem, clone RSO34, 1:200), anti‐p62 mouse monoclonal antibody (BD Biosciences, clone 3/P62 LCK LIGAND, 1:250). Quantification of muscle fibers atrophy and hypertrophy was performed as previously described [7]. Electron microscopy was conducted on muscle fixed with 2.5% glutaraldehyde, 2% paraformaldehyde in 0.1 M cacodylate buffer pH 7.4.

A gene panel for distal and myofibrillar myopathies was done with next generation sequencing on 18 genes (*ACTA1, BAG3, CRYAB, DES, DNAJB6, DNM2, FHL1, FLNC, GNE, HSPB1, HSPB8, MYH2, MYOT, RYR1, SQSTM1, TTN, VCP*, and *ZASP*). Confirmation of the *MYOT* duplication for the proband and analysis for the relatives were done using an MLPA kit (P048‐C3, MRC Holland). A SNP analysis was performed according to routine standard protocol.

Long‐read genome HiFi sequencing was performed for the proband using SMRT sequencing technology (Pacific Biosciences, Menlo Park, CA, USA) on the PacBio Sequel IIe using a custom workflow based on standard methods, as previously described [8, 9]. The sequences were aligned to the GRCh38/Hg38 genome using pbmm2 v.1.4.0. Small variants were phased using Whatshap v.1.1.0 [10] and annotated using a pipeline based on the Variant Effect Predictor v.91 and Gencode 34 basic gene annotations. Short tandem repeat (STR) calling was performed using Tandem Repeat Genotyper (TRGT) v.0.3.3 at 56 previously described disease‐associated STR loci [8, 11]. Structural variant calling was performed using PBSV v. 2.4.0, and the variants were annotated using AnnotSV v.3.1.1 [12].

Western blot was performed in total muscle lysate from patient 1 and pooled healthy controls, using anti‐MYOTILIN rabbit polyclonal antibody (Proteintech 10731‐1‐AP). Post‐transfer myosin was used as loading control.

## RESULTS

### Clinical findings

**FIGURE 1:**
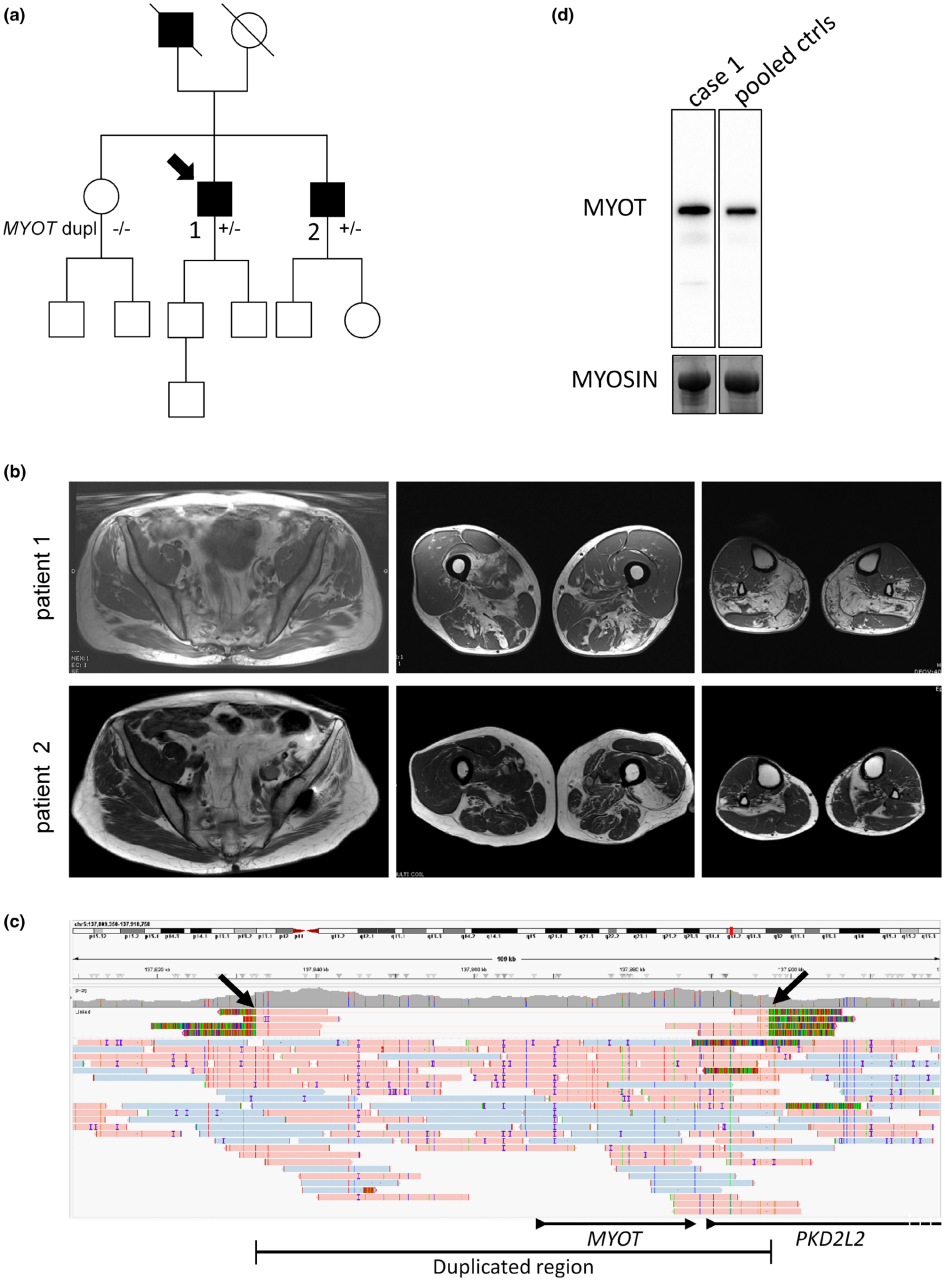
Molecular characterization and muscle imaging phenotype. (a) Pedigree of the family. Clinically affected patients are shown in black. Only the proband and his brother and sister could be tested for *MYOT* gene analysis. (b) Muscle MRI. In the proband (patient 1), severe fatty replacement is present in small glutei in the pelvis. In the thigh, fatty replacement shows a patchy asymmetrical distribution in posterior more than in anterior compartments, with severe involvement of adductor muscles, more patchy and asymmetrical involvement in biceps femoris, semimembranosus and semitendinosus. In the legs, soleus, medial gastrocnemius and peroneal muscles are involved. In the brother (patient 2), fatty replacement is present in small glutei bilaterally and middle and large gluteus on the left side. In the thigh patchy involvement of the left vastus lateralis and adductor muscles is present, while in the legs both soleus and to a lesser extent gastrocnemius are affected. (c) IGV screenshot of the proband's long‐read sequencing results of the duplicated region, including the entirety of *MYOT* as well as part of *PKD2L2*. The phased (haplotyped) alleles are shown in light blue and pink. The reads supporting the duplication (4 out of 20 and 4 out of 17 reads spanning the upstream and downstream breakpoints of the duplication, respectively) are shown in linked mode at the top of the alignment view. These supporting reads are characterized by their stretches of “rainbow” bases (arrows), which indicate that the reads, for those parts, map to another location in the genome than expected. By investigation of the sequences represented by these reads, the duplication was concluded to be in tandem. (d) Western blot analysis of MYOT protein expression of total muscle lysate of patient 1 compared to a pool of healthy controls. Myosin stained by Coomassie is the loading control. The patient and control pool were run on the same gel, the image was cropped for greater clarity.

The proband (patient 1) is a 70‐year‐old man presenting with loss of running ability, difficulty climbing stairs, and calf myalgias since the age of 67. His deceased father presented a similar gait impairment since the age of 70 (Figure 1a). Neuromuscular examination showed an overall hypertrophic appearance in the trunk and upper extremities contrasting with relatively atrophic muscles in the legs. Walking on heels was possible but impossible on tiptoes. Muscle strength was selectively decreased at the level of foot plantar flexion, with weakness corresponding to 3/5 MRC. Gower's sign was negative. Deep tendon reflexes were normal at the upper extremities and decreased at the lower extremities. Mild contracture was present at the level of Achille's tendons. There was no sensory loss. CPK was mildly increased at 371 UI/L (normal values 30–200). ECG and echocardiography were normal. EMG showed decreased compound motor action potentials associated with spontaneous rhythmic discharges at both medial gastrocnemius muscles. These findings were initially interpreted as neurogenic or, more likely, as pseudoneurogenic abnormalities since no high amplitude MUPs was found, making a chronic neurogenic cause unlikely.

Follow‐up neuromuscular assessment showed progressive impairment in walking speed, proximal thigh strength and capacity to stand from a supine position (Table S1).

His older brother (patient 2) had no subjective neuromuscular symptom. His past medical history was significant for left hip arthrosis surgery, lumbar stenosis surgery, and bilateral cataract surgery at the age of 61, retinitis pigmentosa, and right bundle heart block. Neuromuscular examination showed an overall hypertrophic appearance at the trunk and upper extremities without notable other clinical abnormalities except for left psoas muscle weakness (2/5 MRC) and absent tendon reflexes at the lower extremities. He completed 10‐m walk test in 9.1 s. Gower's sign was positive. CPK was normal. EMG showed mild neurogenic changes in left tibialis anterior likely secondary to associated L5 radiculopathy.

### Muscle MRI

Muscle MRI shows fatty replacement of small glutei and both thigh and leg muscles, with greater involvement of posterior than anterior compartments and with prominent bilateral involvement of soleus muscles in both patients (Figure 1b). This pattern is overall similar in both patients, but more limited and more asymmetrical in the older subjectively asymptomatic brother (Figure 1b). Altogether, the clinical‐radiological data are consistent with a late‐onset distal myopathy.

### Genetics

Next‐generation sequencing with a gene panel including distal and myofibrillar myopathies showed a novel heterozygous interstitial duplication of the whole *MYOT* gene not reported so far in control populations (Database of Genomic Variants). However, two CNV gains involving *MYOT* and other genes in gnomAD v4 SV are reported, but their interpretation is challenging due to lack of phenotypic correlates. CGH analysis confirmed the presence of a duplication encompassing the entire *MYOT* gene and the first part of *PKD2L2*. His brother, who had abnormal muscle MRI of the lower extremities, similarly carried the same *MYOT* duplication identified by MLPA, while the sister, who was not carrier of the duplication, had normal muscle MRI. In order to define the orientation of the duplication, long‐read PacBio HiFi sequencing was performed proving the duplication to be in tandem (Figure 1c) and further refining the breakpoints (chr5: 137,832,296–137,897,203dup). No other putative disease‐explanatory small variants, STR expansions, or structural variants were identified in the long‐read sequencing data. To understand whether this duplication altered MYOT protein expression we performed a Western blot analysis in muscle of patient 1. Compared to a pooled group of healthy controls, MYOT expression seemed to increase (Figure 1d). Although quantitative statistics cannot be provided, this observation is compatible with a gene dosage effect on MYOT expression.

### Muscle pathology

**FIGURE 2:**
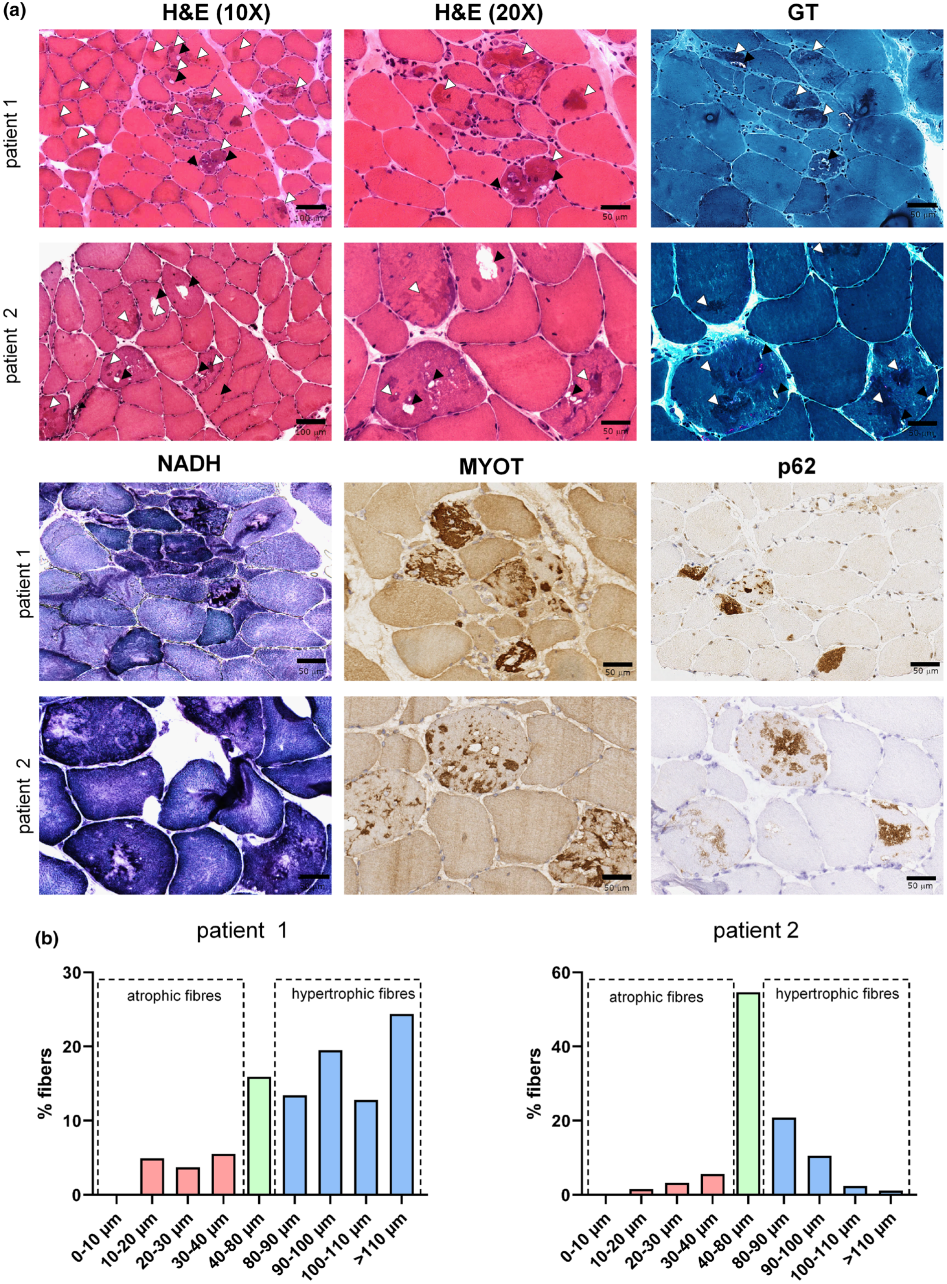
Myopathological findings. (a) Light microscopy. Hematoxylin and eosin (H&E) staining shows increased fiber size variability with many hypertrophic fibers and some atrophic fibers, sometimes with an angular appearance. Some central nuclei are present and connective tissue content is increased. Cytosolic vacuoles, mostly non‐rimmed are indicated by black arrows. Basophilic material is evident in several fibers (white arrows). Similar findings are evident with Gomori trichrome staining (GT). NADH‐tetrazolium histoenzymatic reaction (NADH) shows abnormal myofibrillar network organization in both cases, with areas of variable size lacking reaction with core‐like lesions, and focal areas corresponding to the amorphous material indicated above showing intense reaction. Staining with anti‐myotilin antibody shows intense reaction of the protein deposits above described (MYOT). Strong reaction with anti‐p62 antibody in some muscle fibers. (b) Quantification of atrophic and hypertrophic muscle fibers. Atrophy factor is 274 for case 1 (*n* = 164 fibers analyzed) and 172 for case 2 (*n* = 533 fibers analyzed); hypertrophy factor was abnormally increased to 1884 for case 1 and 536 for case 2 [7].

**FIGURE 3:**
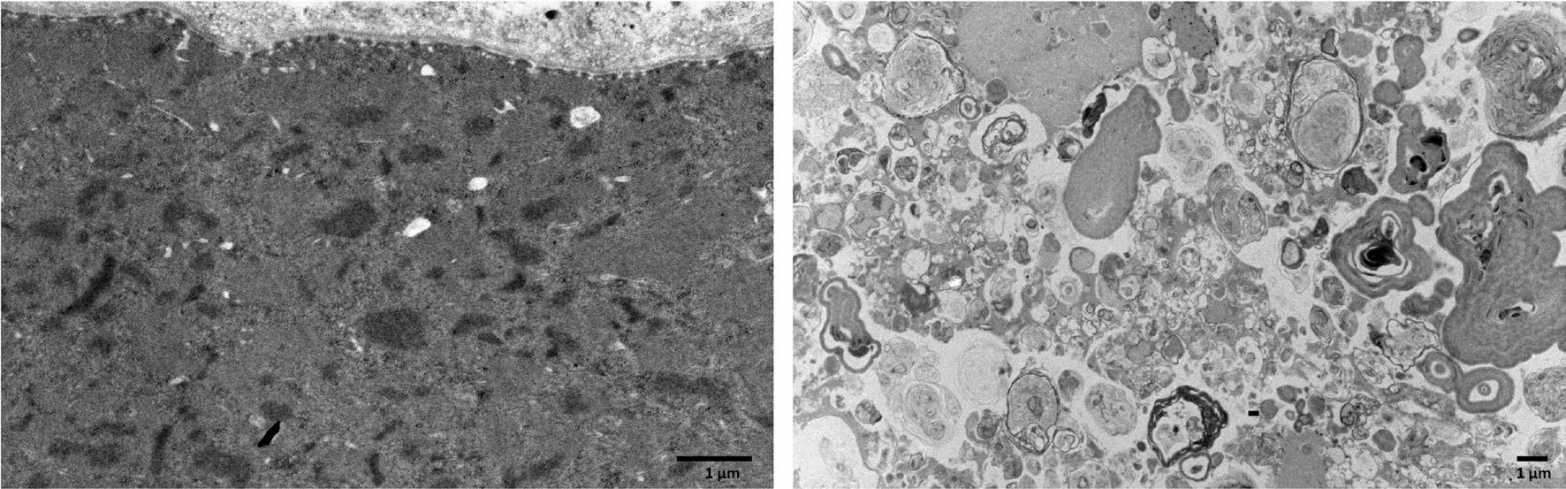
Electron microscopy. Destruction of myofibrillar organization with accumulation of material derived from Z‐disk (left). Severe autophagic abnormalities and accumulation of degraded cytosolic material (right).

To validate the pathogenicity of this novel *MYOT* duplication we performed a detailed myopathological analysis of muscle biopsies looking for evidence in support of myotilinopathy. Light microscopy examination of the muscle biopsy of both patients showed the presence of central nuclei in some fibers, increased fiber size variability with the coexistence of hypertrophic muscle fibers and atrophic fibers, increased collagen tissue, myofibrillar disarray, large cytoplasmic rimmed and non‐rimmed vacuoles, and amorphous protein aggregates (Figure 2a). Few fibers were necrotic. ATPase staining showed small foci of type grouping and focal areas of reaction depletion within some fibers corresponding to the areas of pathological protein aggregates. HLA1 and 2 were negative and no inflammatory infiltrates were present. Protein aggregates were weakly positive for Desmin but strongly reactive with anti‐Myotilin antibody (Figure 2a). Moreover, protein aggregates showed p62 (Figure 2a) and partially by anti‐TDP43 antibody immunoreactivity. Vacuoles were positively stained by dystrophin (data not shown). To evaluate more precisely fiber size variability, we performed a quantitative analysis of fiber atrophy and hypertrophy factors. Both hypotrophic and hypertrophic fibers were present in both cases; however, hypertrophic fibers were more frequent than atrophic fibers (Figure 2b). Electron microscopy examination showed areas of focal myofibrillar disarray, signs of lysosomal/autophagic abnormalities including rimmed vacuoles (Figure 3) and lipofuscin, and occasional tubular aggregates.

## DISCUSSION

Myofibrillar myopathies are very rare inherited myopathies with heterogeneous clinical phenotypes and genetic causes [2]. In France, desminopathy is the most frequent type of MFM, while myotilinopathy is quite rare, with only 8 patients identified among 76 patients with a molecular diagnosis of MFM identified in Paris and Marseille [2]. In Italy, myotilinopathy is the second most common genetic cause [13]. The clinical, radiological, and pathological features of this family fall into the known phenotypic spectrum of myotilinopathies [2, 4, 14], clinically characterized by a late‐onset asymmetrical distal‐proximal myopathy of the lower limbs associated with upper body muscle hypertrophy, and pathologically by myotilin aggregates in skeletal muscle associated with autophagic vacuoles with sarcolemmal features. Muscle hypertrophy was paralleled with frequent muscle fibers hypertrophy in our cases. In one of the two patients the clear muscle pathology had not yet resulted in clinical symptoms perceived by the patient, which is line with previous observations of variable disease penetrance and severity [2]. This is also consistent with previous studies reporting a considerable proportion of cases as “sporadic” [15]. Therefore, it is important for clinicians to bear this consideration in mind to avoid underdiagnosis or misdiagnosis with peripheral neuropathy. The presence of variable levels of muscle hypertrophy can be misleading and lead overlooking the diagnosis of myopathy. Misdiagnosis with peripheral neuropathy can occur due to the common and sometimes exclusive distal involvement at the lower extremities, coexistence with peripheral neuropathy [16], and the common presence of abnormal spontaneous activity in rimmed vacuolar myopathies including myotilinopathies and other myofibrillar myopathies such as *GNE* and *PLIN4* [13] as well as Pompe disease [17] that can be misinterpreted for active denervation [2]. Spontaneous rhythmic activity, as found in our proband, is likely caused by muscle involvement. A differential diagnosis with inclusion body myositis should also be considered, which shares with myotilinopathy the late onset of disease, distal muscle involvement, common pseudoneurogenic EMG abnormalities, and rimmed vacuole pathology. However, different distal muscles are usually involved in the two conditions, and the coexistence of both inflammatory features and severe mitochondrial dysfunction allows for differentiating these two diseases. Importantly, there is significant clinical, genetic, and myopathological overlap between MFM and distal myopathies, since a distal presentation is common in MFM including myotilinopathy, and rimmed vacuoles can be found in both distal myopathies and MFM [13]. Therefore, clinicians should consider both MFM and distal myopathies in addition to peripheral neuropathies in patients presenting with distal muscle weakness, even in the elderly.

Most reported cases with myotilinopathy carry one of two common missense mutations, p.S55F, and p.S60F [2, 4] likely originating from ancient founder effects. The vast majority of patients are heterozygous *MYOT* mutation carriers, more rarely they are homozygous carriers and both heterozygous and homozygous patients can coexist in the same family [18]. We report here the first family of myotilinopathy caused by a duplication of the *MYOT* gene. Our clinical, pathological and molecular findings in the presented family are consistent with a diagnosis of myotilinopathy [3, 4, 15, 19], providing evidence for pathogenicity of the novel duplication. This suggests that the gene dosage of *MYOT* may be important to maintaining the physiological integrity of the Z‐disk structure. Interestingly, overexpression of WT MYOT in mice leads to moderate aggregation of MYOT and associated proteins but not muscle degeneration during the relatively short life span of mice [20]. Here, we show that duplication of the whole *MYOT* gene leads to increased expression and aggregation of MYOT causing late‐onset myopathy. This observation is consistent with the requirement of precise stoichiometry of Z‐disk components such as myotilin and Filamin‐C [21]. Similar cases of gene dosage have been reported in other neurological diseases. In Charcot–Marie–Tooth Disease Type 1A (CMT1A), a common duplication of the *PMP22* gene leads to overproduction of the PMP22 protein, which disrupts the structure and function of myelin in peripheral nerves, resulting in neuropathy [22]. In Pelizaeus‐Merzbacher Disease (PMD), duplication of the *PLP1* gene results in excess PLP1 protein, which can misfold and aggregate, impairing the formation of myelin sheaths in the central nervous system [23]. Pathological protein aggregation is also a common mechanism of common neurodegenerative diseases such as Parkinson's disease and Alzheimer's disease. Interestingly, in Down syndrome, patients have an extra copy of chromosome 21, which includes the *APP* gene. This leads to the overexpression of APP, increasing the production and aggregation of amyloid‐beta (Aβ) peptides, which are crucial in the pathogenesis of Alzheimer's disease [24]. Interestingly, amyloid‐beta has been reported to accumulate in muscle of patients with myofibrillar myopathies [16].

Although the pathophysiological mechanisms of myotilinopathies are not still completely understood, they likely imply both protein toxicity leading to protein aggregation, and loss of function effects due to sequestration of Myotilin and other Z‐disk components in the aggregates, opposed to depletion of the same proteins in surrounding areas [21]. Myotilin aggregation leads to downstream destruction of Z‐disk assembly and accumulations of associated proteins, activation of autophagy, upregulation of chaperones and unfolded protein response, as well as remodeling of cytoskeleton [21].

In conclusion, we report here a whole gene *MYOT* duplication causing late onset myotilinopathy, possibly through a gene dose effect. A high level of awareness is crucial to reach the diagnosis since the clinical phenotype may be mild and easily overlooked or misdiagnosed as distal motor neuropathy. The application of long‐read genome sequencing is helpful in the identification and definition of duplicated and repetitive genomic regions linked to genetic diseases.

## AUTHOR CONTRIBUTIONS

**Marco Spinazzi:** Conceptualization; investigation; funding acquisition; writing – original draft; methodology; validation; visualization; formal analysis; data curation; supervision; writing – review and editing. **Marco Savarese:** Investigation; validation; formal analysis; data curation; writing – review and editing. **Franck Letournel:** Investigation; writing – review and editing. **Lydia Sagath:** Writing – review and editing; investigation; methodology. **Florence Manero:** Methodology; writing – review and editing. **Agnès Guichet:** Investigation; writing – review and editing. **Alexander Hoischen:** Investigation. **Corinne Metay:** Investigation; writing – review and editing. **Julien Gouju:** Investigation; writing – review and editing; data curation. **Bjarne Udd:** Validation; writing – review and editing; data curation; formal analysis; investigation.

## FUNDING INFORMATION

This work has been partially supported by AFM‐Telethon to MS (25065). MS is supported by INSERM, being recipient of an INSERM translational research grant (CIHU INSERM).

## CONFLICT OF INTEREST STATEMENT

The authors declare no conflict of interest.

## Supporting information


Table S1.

## Data Availability

The data that support the findings of this study are available from the corresponding author upon reasonable request.
